# Recognition of Empathy from Synchronization between Brain Activity and Eye Movement

**DOI:** 10.3390/s23115162

**Published:** 2023-05-29

**Authors:** Jing Zhang, Sung Park, Ayoung Cho, Mincheol Whang

**Affiliations:** 1 International Research Center of Architecture and Emotion, Hebei University of Engineering, Handan 056009, China; zj03010020@gmail.com; 2 Department of Emotion Engineering, Sangmyung University, Seoul 03016, Republic of Korea; sjpark@smu.ac.kr (S.P.); joa6391@gmail.com (A.C.); 3 Department of Human-Centered Artificial Intelligence, Sangmyung University, Seoul 03016, Republic of Korea

**Keywords:** brain activity, digital content, EEG, empathy, eye movement, physiological measures

## Abstract

In the era of user-generated content (UGC) and virtual interactions within the metaverse, empathic digital content has become increasingly important. This study aimed to quantify human empathy levels when exposed to digital media. To assess empathy, we analyzed brain wave activity and eye movements in response to emotional videos. Forty-seven participants watched eight emotional videos, and we collected their brain activity and eye movement data during the viewing. After each video session, participants provided subjective evaluations. Our analysis focused on the relationship between brain activity and eye movement in recognizing empathy. The findings revealed the following: (1) Participants were more inclined to empathize with videos depicting pleasant-arousal and unpleasant-relaxed emotions. (2) Saccades and fixation, key components of eye movement, occurred simultaneously with specific channels in the prefrontal and temporal lobes. (3) Eigenvalues of brain activity and pupil changes showed synchronization between the right pupil and certain channels in the prefrontal, parietal, and temporal lobes during empathic responses. These results suggest that eye movement characteristics can serve as an indicator of the cognitive empathic process when engaging with digital content. Furthermore, the observed changes in pupil size result from a combination of emotional and cognitive empathy elicited by the videos.

## 1. Introduction

Empathy is the capacity to understand and resonate with the experiences of others, and can depend on the ability to predict when others are likely to receive rewards [1]. Preston and de Waal proposed a neuroscientific model of empathy, showing that observing or imagining another person in a particular emotional state automatically activates the observer’s representation of a state and its associated autonomic and somatic responses [2]. Empathy comprises two processes: cognition and emotion. Cognitive empathy involves the ability to understand how others perceive experiences through their senses such as sight or hearing. Emotional empathy, on the other hand, relates to sensitivity towards others’ emotions. Key neural components of affective arousal include subcortical circuits such as the amygdala, hypothalamus, hippocampus, and orbitofrontal cortex (OFC).

The amygdala and OFC, both connected reciprocally with the superior temporal sulcus (STS), play a vital role in the rapid and prioritized processing of emotional signals. This processing largely coincides with theory-of-mind-like processing, which relies on the ventromedial (VM) and medial (M) prefrontal cortex (PFC) and involves executive functions. Emotion regulation allows for control over emotions, affects, drives, and motivations. The dorsolateral PFC, anterior cingulate cortex (ACC), and vmPFC are primarily responsible for self-regulation through their reciprocal connections with the amygdala and extensive cortical areas including the STS. As a result, empathy is not merely a passive affective resonance phenomenon involving the emotions of others. Instead, factors such as goals, intentions, context, and motivations play proactive roles in shaping the perception and experience of emotions [3].

The functional neuroanatomy of empathy divides empathy research into six types including mirror neurons and theory of mind [4]. Mirror neurons are cells that respond to both movement and the observation of others’ actions. These neurons seem to connect input directly to output, allowing us to understand others’ behavior by comparing it to our own. Furthermore, mirror neurons enable us to relate what others say directly and effectively to our mechanisms for generating speech [5].

Mirror neurons are located in the premotor cortex, parietal lobe, and the anterior insula of the temporal lobe [6]. The frontal lobe, which is the most recently evolved area of the brain, plays a significant role in empathy. The parietal lobe is a high-level region responsible for integrating vision, hearing, and body perception. It is also noteworthy that the F5 region, which houses mirror neurons, is a homolog of Broca’s region, which is responsible for language [7]. Empathy goes beyond mere emotional contagion or imitation. Neuroscientific evidence indicates that empathy does not solely result from passive observation of emotional cues, but is also influenced by contextual evaluation and regulation [3].

There is now considerable evidence that brain regions that were initially considered higher up in a processing hierarchy can modulate lower regions through so-called re-entrant processing from descending neural pathways, and these sorts of modulation are often also commonly called top-down effects [8,9,10].

In the case of vision, the distinction between the center of consciousness and the periphery becomes evident. The center of consciousness involves focused visual attention, while the periphery encompasses areas that are less focused but still within the realm of conscious awareness. Vision within the central area of consciousness is predominantly conscious in nature [11]. Vision comprises both unconscious visual information directed towards the parietal lobe and conscious visual data relayed to the temporal lobe. Consequently, sensory consciousness serves as an appropriate medium for exploring consciousness through the comparison of conscious and unconscious states [12].

Visual processing is divided into unconscious visual processing from the primary visual cortex to the parietal lobe, and conscious visual processing from the temporal lobe [13]. Visual processing in the parietal lobe involves several unconscious movements that trigger hand movements [14]. Going step-by-step regarding the hand gesture for grabbing the cup, first with the palm open and then moving closer to the cup, the fingers are subconsciously and adequately pulled inward to catch the round coward. The ability to automatically adjust many cotton movements in response to recognizing objects by sight is a visual process that is processed in the parietal lobe. The primary visual cortex processes line segments, angles, and edges, and v2 performs further analysis using information input from v1. v4 handles color, and MT controls movement [15].

Visual information is transmitted from the primary visual cortex to the inferior temporal lobe, where the shape and color of an object are processed to form a memory of a distinct object [16]. Visual perception generated in the inferior temporal lobe is then linked to the frontal lobe, becoming conscious. The two streams of visual information are interconnected through the sagittal pathway, which involves the frontal visual cortex. This cortex is connected to the dorsolateral prefrontal and orbitofrontal cortices, allowing for attention to be focused or diverted based on visual input. The ability to shift attention from one object to another offers continuity and adaptability to cognitive processes [17].

In a study by Zheng et al., EEG and eye-tracking signals were used for emotion recognition, demonstrating that the combination of these two data types can enhance the performance of emotion recognition modules [18]. Similarly, Lu et al. developed a multimodal emotion recognition framework that combined eye movement and electroencephalography (EEG) to improve emotion recognition accuracy. Experimental results indicated that fusing multiple modalities significantly increased the precision of emotion recognition compared to using a single modality [19]. Thus, eye movement information can provide valuable data that aids in the identification of human emotional states [20,21].

We are interested in examining the state of empathy, in which visual perception information alternates between the brain’s conscious and unconscious processing. Moreover, we aim to investigate the arousal of cognitive and affective empathy by studying changes in brain activity, the responses of these changes within the central nervous system, and the autonomic feedback state of the nervous system as it pertains to the eyes. This paper employs the synchronization of brain activity states and alterations in eye movement features to explore the components of empathy, as well as the changes in brain activity and eye movement when cognitive and affective empathy are elicited.

## 2. Materials and Methods

### 2.1. Stimuli Selection

We edited video clips (dramas or movies) to elicit empathy from participants. The content that induced empathic conditions was collected using a two-dimensional model. We conducted a stimulus selection experiment before the main experiment to ensure that the empathic and non-empathic videos were effective. We selected 20 edited dramas or movies containing emotions as stimuli. Five video clips for each quadrant (pleasant-aroused, pleasant-relaxed, unpleasant-relaxed, and unpleasant-aroused) in a two-dimensional model were selected. Thirty participants viewed the videos and responded to a subjective questionnaire. Each participant received $20 for study participation. For each quadrant, among the five candidates, the video with the highest empathic score was selected as the empathic stimulus for the main experiment. Conversely, the video with the lowest empathic score was chosen as the non-empathic stimulus. In summary, a pair of empathic and non-empathic videos were selected for each of the four quadrants in the two-dimensional model. Eight stimuli were selected for the main experiment.

### 2.2. Experiment Design

When the observer is interested in the target stimulus, the observer’s eye movement changes because of the emotional characteristics of the target (empathy, valence, and arousal). The main experiment was designed to understand the relationship between these changes and the brain activity. This was a factorial design of two (empathy: empathic and non-empathic) × two (valence: pleasant and unpleasant) × two (arousal: aroused and relaxed) independent variables. We measured participants’ brain activity in major regions (frontal lobe, parietal lobe, temporal lobe, occipital lobe, and central lobe) and eye movement features (left and right pupil size, gaze, and saccade).

### 2.3. Participants

We conducted an a priori power analysis using the program G*Power 3.1 with the power set at 0.8 and α = 0.05, d = 0.6 (independent *t*-test), two-tailed. Adopting a conservative approach, we powered our study to detect a moderate effect size (Cohen’s d = 0.6) [22], taking into account the context of neuroscience studies [23]. These results suggest that an N value of approximately 46 is required to achieve appropriate statistical power. Therefore, 47 university students were recruited for the study. The study analyzed the data acquired from the same participant in a different study, which investigated the relationship between brain connectivity and eye movement features [24]. The previous study compared the means of different groups.

Participants’ ages ranged from 20 to 30 years (mean = 28, STD = 2.9), with 20 (44%) men and 27 (56%) women. We selected participants with a corrective vision ≥0.8 without any vision deficiency to ensure reliable recognition of visual stimuli. We recommended that the participants sleep sufficiently and prohibited alcohol, caffeine, and smoking the day before the experiment. Because the experiment required valid recognition of the participant’s facial expression, we limited the use of glasses and cosmetic makeup. All participants received a briefing regarding the purpose and procedure of the experiment and signed a consent form. They were then monetarily compensated for their participation.

### 2.4. Experimental Protocol

**Figure 1:**
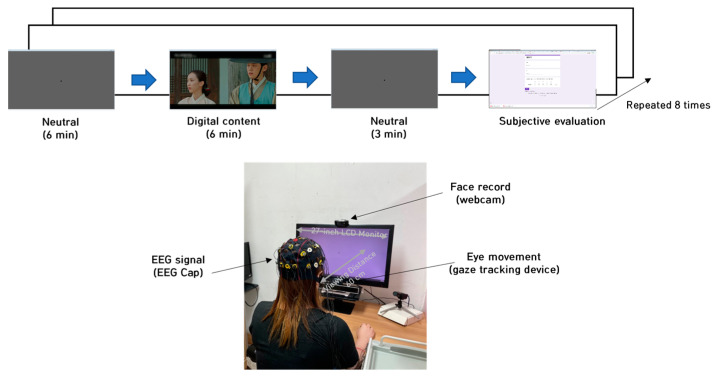
Experimental protocol and environment.

Figure 1 depicts the experimental process and environment for the study. The participants were asked to sit 1 m away from a 27-inch LCD monitor. A webcam was installed on the monitor. The participants viewed eight emotion-eliciting (empathy or non-empathy) videos and responded to a questionnaire after each viewing. During each viewing, participants’ brainwaves (EEG cap 18 ch), facial expressions (webcam), and eye movements (gaze tracking device) were acquired. The facial expression data have been examined in a separate study [24] and are not included in the analysis for this particular research. Eye movements and pupil size were recorded at 60 Hz using a GazePoint device (GazePoint GP3 Inc., Vancouver, BC, Canada). The EEG signals were recorded at a 500 Hz sampling rate from 18 channels (Fp1, Fp2, F3, F7, Fz, F4, F8, T3, T4, C3, C4, T5, P3, Pz, P4, T6, O1, and O2 regions) based on the international 10–20 system (ground: Cz; DC level: 0–150 Hz) using an EEG device (202, Mitsar Inc., St. Petersburg, Russia).

The fixation measures represent the distance between the two eye fixations (i.e., fixations A and B). The raw data were obtained from the squared root of the sum of squared fixation coordination A and squared fixation coordination B. The raw data were filtered using a band-pass filter. For example, fixation between 1 and 4 Hz represents the degree of location change between 0.25 and 1 s. Saccade measures represent the degree of change between the two fixation points divided by time. We used the velocity-threshold identification fixation classification algorithm to record saccades when the gaze position is above a certain threshold, as a function of time.

We collected participants’ subjective responses using the consumer empathic response to advertising scale (CERA) (see Table 1), a comprehensive set of measures that encompass both affective and cognitive aspects of empathy [25,26,27].

### 2.5. Assessment of Brain Activity from Eye Movement and Pupillary Response

EEG signals were processed using a band-pass filter (BPF) of 1–50 Hz, and the EEG spectrum was analyzed using the fast Fourier transform (FFT) method. The EEG spectrum was divided into the following ranges according to the frequency band: delta 1–4 Hz; theta 4–8 Hz; alpha 8–13 Hz; beta 13–20 Hz [28,29]. Eye movement and pupil size were processed using BPF into separate frequency bands: delta (0.12–0.48 Hz), theta (0.48–0.96 Hz), alpha (0.96–1.56 Hz), and beta (1.56–3.6 Hz). For synchronization, we applied a 60/500 ratio between the eye movements and EEG signals. Each band power was defined by summing the frequency power values from each spectrum data. The relative power of each frequency band, from delta to beta, was calculated using the ratio between the total and each band power, as shown in Equation (1).

(1) $$each\;band\;ratio=\frac{each\;band\;power}{total\;power}$$

**Figure 2:**
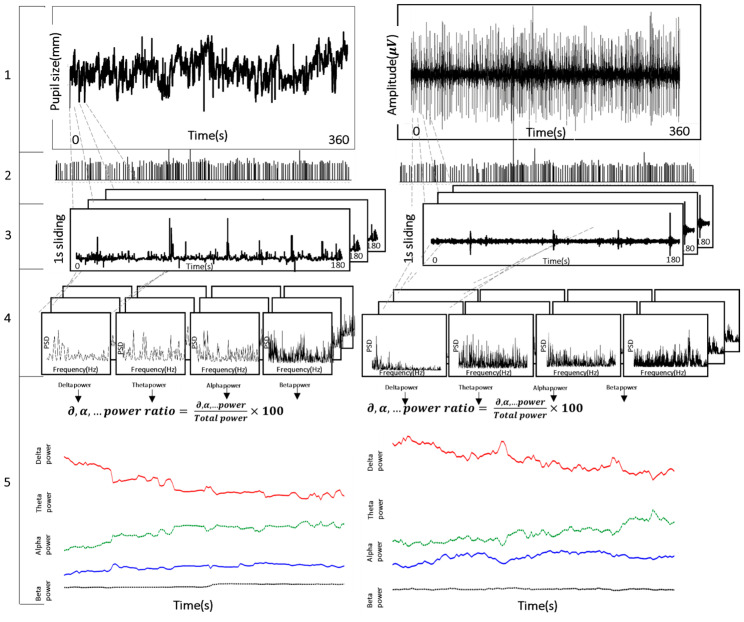
The five steps of the synchronization process between eye movement (**left**) and brain activity (**right**) in analysis.

Figure 2 outlines the synchronization process adopted in [30]. The process consisted of five consecutive steps: (1) Sampling EEG at 500 Hz and eye movement at 60 Hz. (2) Removing blinks. (3) Applying a window size of 180 s and a sliding size of 1 s. (4) Band-pass filtered signals of each frequency band and power spectral density (PSD) for each band from the feature. (5) Each band ratio was calculated using the ratio between the total power and each band power.

### 2.6. Synchronization with Brain Activity from Eye Movement and Pupillary Response

**Figure 3:**
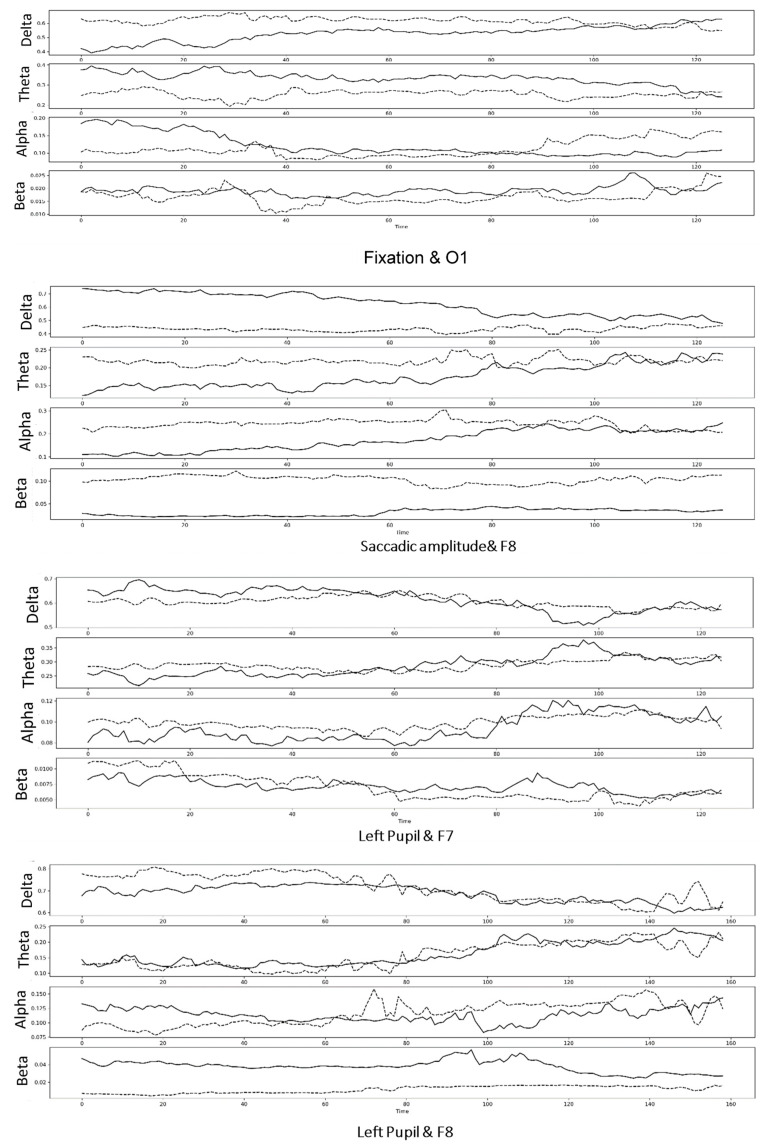
Four instances of synchronization of an EEG and eye movement feature as a function of time. The dotted line represents eye movement feature, and the solid line represents EEG signals.

Figure 3 represents four instances where synchronization between an EEG and eye movement features occurred, specifically between eye fixation and O1, saccadic amplitude and F8, left pupil and F7, and right pupil and F8. The two features gradually converged as a function of the time spent watching the empathic video. To model synchronization and fit, we conducted regression analysis.

### 2.7. Regression Analysis

We adopted five regression models for analysis: Bayesian ridge, linear regression, elastic net, support vector regression (SVR), and gradient boosting regression (GBR). We mapped eye movement feature measures on the EEG channel using a regression formula. The regression function is y = f(x), where x is the eye movement measure and y is the EEG feature measure. The R^2^ value indicates the prediction percentage (%) when comparing the predicative y measures using a particular regression model to the actual y measures. 

**Figure 4:**
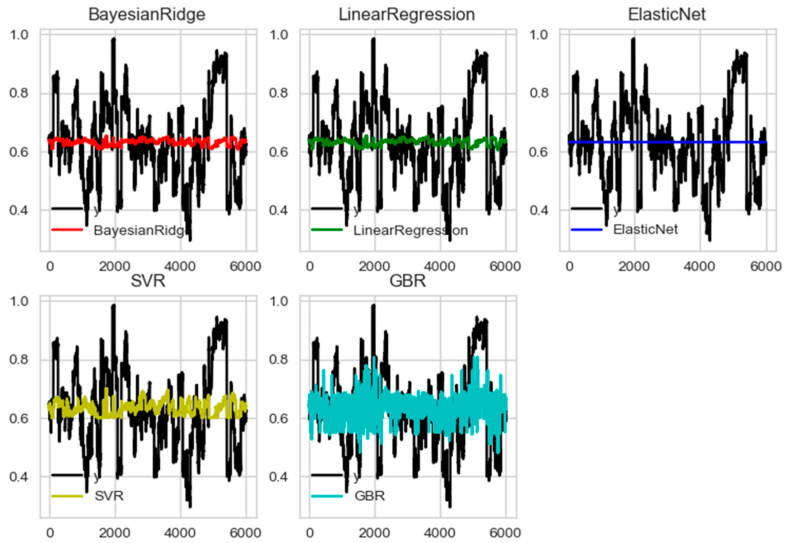
The result of five regression models in the delta frequency domain of the left pupil (eye features) and Fp1 (EEG features). *X*-axis represents time (msec), and the *y*-axis represents accuracy.

Figure 4 shows the regression prediction (colored) overlaid on the delta frequency domain of the left pupil (eye features) and Fp1 (brain activity) under the pleasant-aroused condition as an example. The proportion of the training dataset to the test dataset was 7:3. The following are the hyperparameters of our models:

1. BayesianRidge (alpha_1 = 10^−6^, alpha_2 = 10^−6^, compute_score = False, copy_X = True, fit_intercept = True, lambda_1 = 10^−6^, lambda_2 = 10^−6^, n_iter = 300, normalize = False, tol = 0.001, verbose = False);
2. LinearRegression (copy_X = True, fit_intercept = True, n_jobs = 1, normalize = False);
3. ElasticNet (alpha = 1.0, copy_X = True, fit_intercept = True, l1_ratio = 0.5, max_iter = 1000, normalize = False, positive = False, precompute = False, random_state = None, selection = ‘cyclic’, tol = 0.0001, warm_start = False);
4. SVR (C = 1.0, cache_size = 200, coef0 = 0.0, degree = 3, epsilon = 0.1, gamma = ‘auto’, kernel = ‘rbf’, max_iter = −1, shrinking = True, tol = 0.001, verbose = False);
5. GradientBoostingRegressor (alpha = 0.9, criterion = ‘friedman_mse’, init = None, learning_rate = 0.1, loss = ‘l s’, max_depth = 3, max_features = None, max_leaf_nodes = None, min_impurity_decrease = 0.0, min_impurity_split = None, min_samples_leaf = 1, min_samples_split = 2, min_weight_fraction_leaf = 0.0, n_estimators = 100, presort = ‘auto’, random_state = None, subsample = 1.0, verbose = 0, warm_start = False);

The model performance evaluation measured the gap between the predicted and true values. The most intuitive evaluation indicators include the mean absolute error (MAE) and root mean square error (MSE), as shown in Equations (2) and (3). We also applied the R^2^ evaluation method, which considers the difference between regression and real values, as shown in Equation (4).

(2) $$MAE(X,h)=\frac{1}{m}\sum_{i=1}^{m}\left|{h(x}^{\left(i\right)})-y^{\left(i\right)}\right|$$

(3) $$MSE=\frac{1}{n}\sum_{i=1}^{n}{\left(y_{i}-{\overset{-}{y}}_{i}\right)}^{2}$$

(4) R2=1−∑iyi−y^i2∑i−yi−y¯i2

Table 2 includes the results of each regression analysis. Table 3 outlines the evaluation indicators. Overall, we learned that the GBR model had the best fit based on the regression of the eigenvalues of the EEG and eye movement features. Hence, we decided to use the GBR as a prediction method for further analysis.

## 3. Results

### 3.1. Saccade and Fixation Result

**Figure 5:**
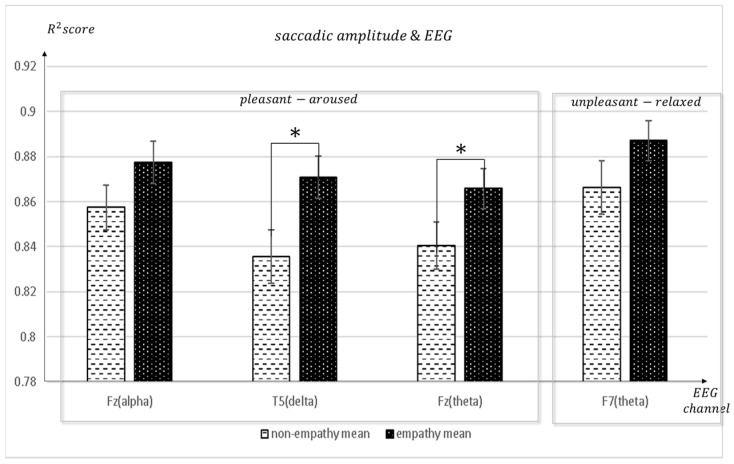
The mean of the R^2^ score from the regression analysis of saccadic amplitude and EEG channels between the non-empathic and empathic conditions. * *p* < 0.01.

**Figure 6:**
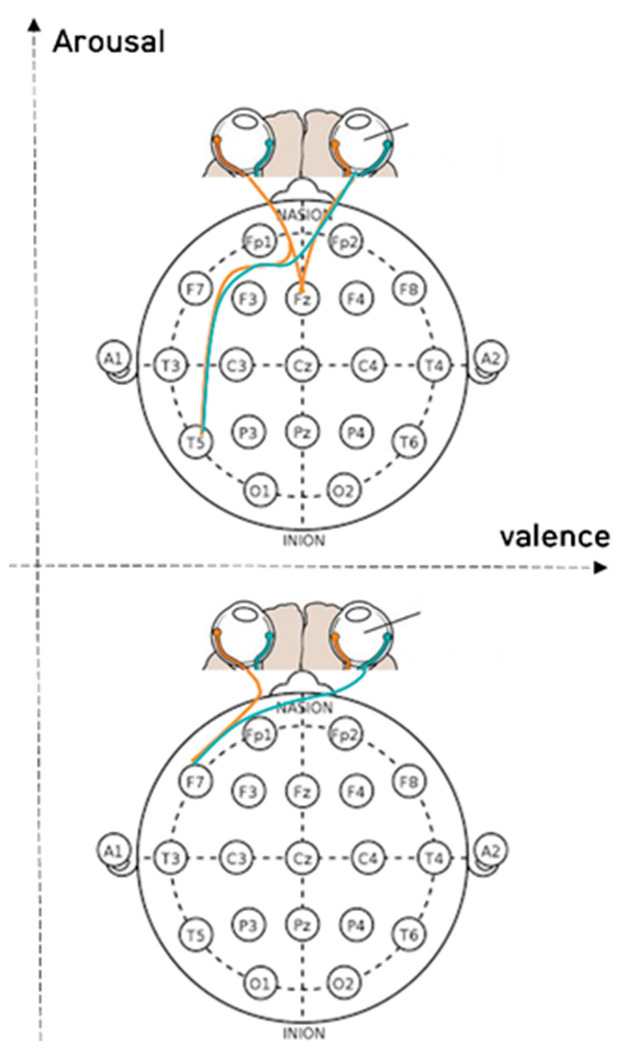
The saccadic amplitude and EEG channels in each frequency band in empathic states.

Figure 5 shows the mean value of the R^2^ scores between empathic and non-empathic conditions when the R^2^ score was obtained from the regression analysis between the saccadic amplitude (eye movement) and EEG measures. The R^2^ score indicates the degree of fit between the GBR prediction and the real data. As the regression results (EEG feature measure) were a function of eye movement, the higher the R^2^, the higher the synchronization between the two measures. Figure 6 depicts the saccadic amplitude and EEG channels of each band in the empathic states. Yellow indicates a connection in the same hemisphere, and green indicates a connection in the crossed hemisphere.

We used the following exclusion criteria to consider EEG-eye movement synchronization when selecting comparison conditions. We excluded the following conditions: (1) the R^2^ value of the non-empathic condition was higher than that of the empathic condition, and/or (2) the standard deviation of each mean overlapped.

In this study, consistent with the subjective evaluation results, pleasant-aroused, unpleasant-relaxed empathy appeared more in the synchronization between the EEG and eye movement. Pleasant-aroused, unpleasant-relaxed recognizes emotions in the video and shows empathy while referring to previous experiences or long-term memories. In the synchronization between EEG and eye movement, viewers showed empathy for the pleasant-aroused video, among which both cognitive and emotional empathy appeared with more cognitive empathy.

**Figure 7:**
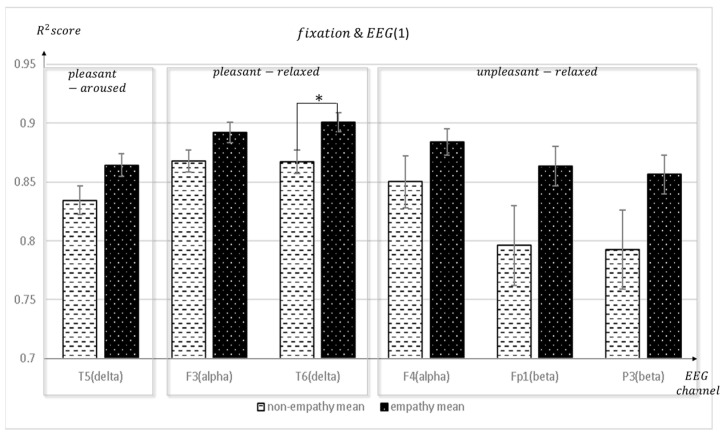
The mean value of the R^2^ score obtained by regression analysis of fixation and EEG channels (T5, F3, T6, F4, Fp1, P3) in each frequency band when empathic states are compared with non-empathic states. * *p* < 0.01.

**Figure 8:**
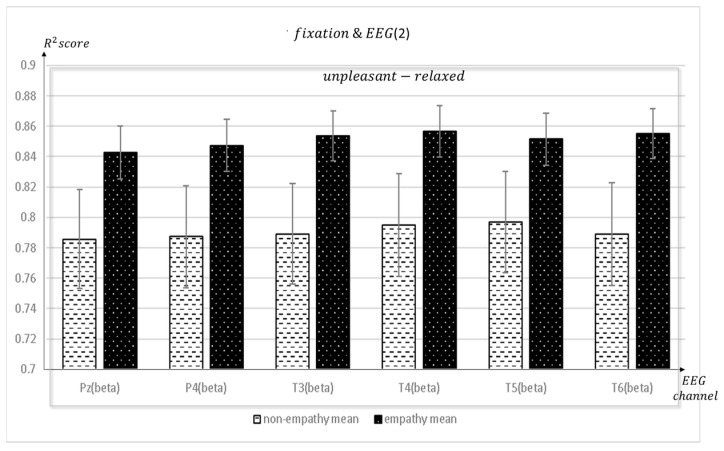
The mean value of the R^2^ score obtained by regression analysis of fixation and EEG channels (Pz, P4, T3, T4, T5, T6) in each frequency band when empathic states are compared with non-empathic states.

**Figure 9:**
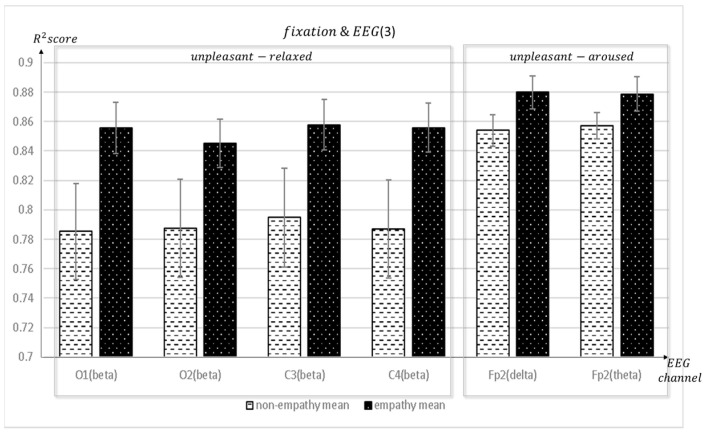
The mean value of the R^2^ score obtained by regression analysis of fixation and EEG channels (O1, O2, C3, C4, Fp2) in each frequency band when empathic states are compared with non-empathic states.

**Figure 10:**
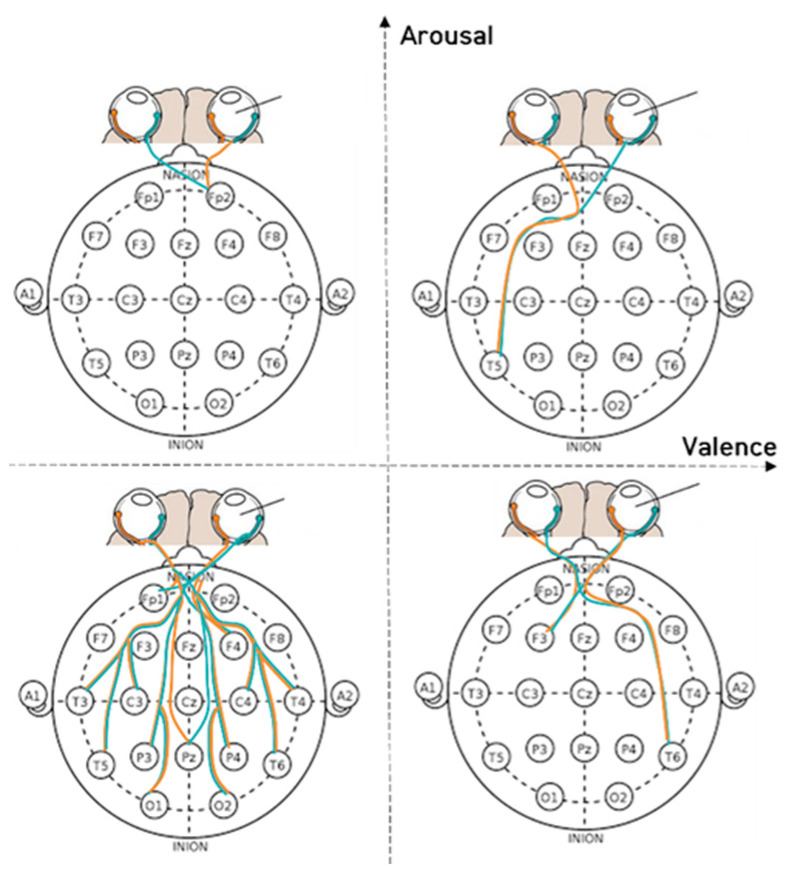
The fixation and EEG channels in each frequency band in empathic states.

Figure 7, Figure 8 and Figure 9 depict the fixation and EEG channel synchronization for each band ratio. Figure 10 depicts the fixation and EEG channels of each band in the empathic states.

### 3.2. Left Pupil and Right Pupil Result

Pupil changes are controlled by the central nervous system (oculomotor nerve) and autonomic nervous system (iris muscle). The fast reflex cortical pathway is involved in emotional contagiousness in the empathic system, and the slow cortex is involved in cognitive empathy. At each stage of consciousness, the unconscious may appear randomly in the form of consciousness.

**Figure 11:**
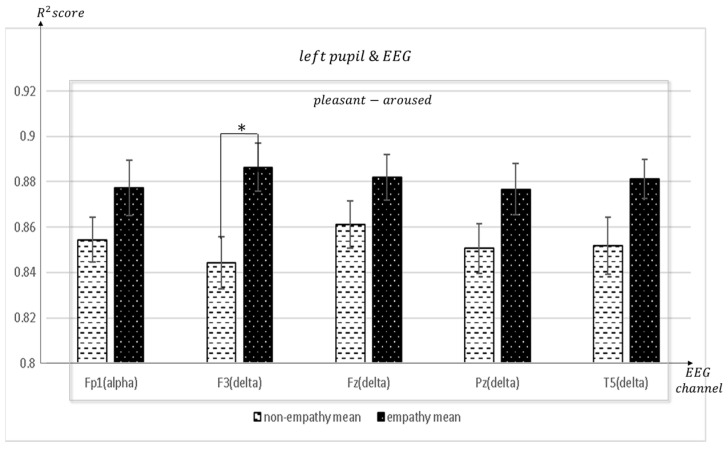
The mean value of the R^2^ score obtained by regression analysis of left pupil and EEG channels in each frequency band when empathic states are compared with non-empathic states. * *p* < 0.01.

**Figure 12:**
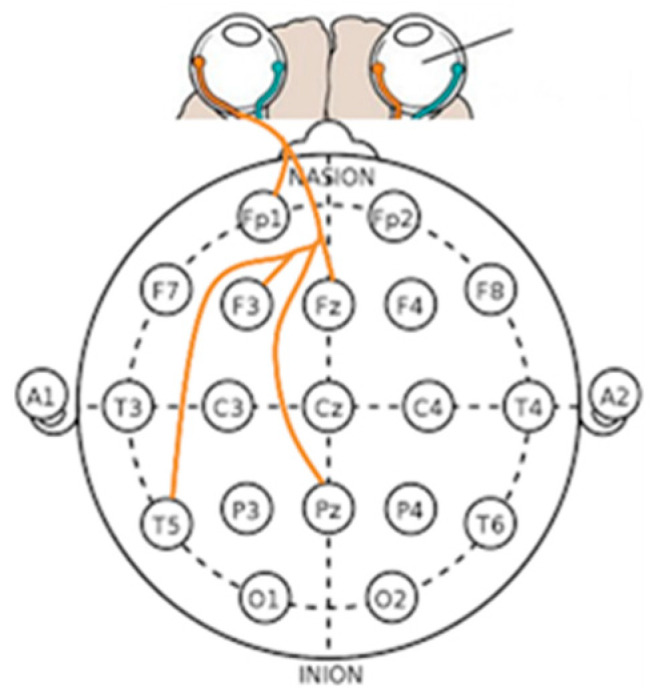
The left pupil and EEG channels in each frequency band in empathic states.

**Figure 13:**
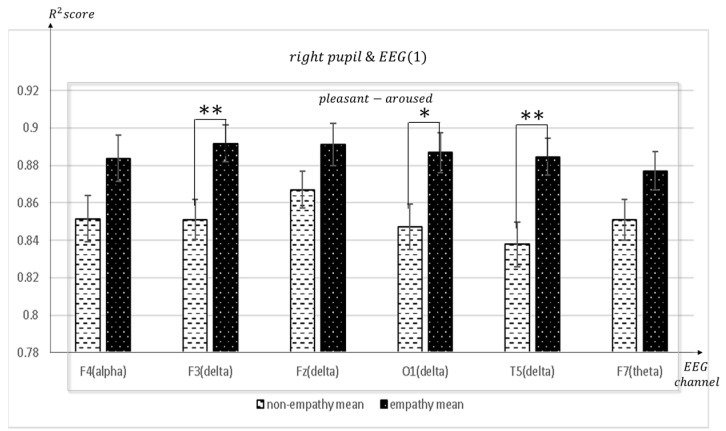
The mean value of the R^2^ score obtained by regression analysis of right pupil and EEG channels (F4, F3, Fz, O1, T5, F7) in each frequency band when empathic states are compared with non-empathic states. * *p* < 0.01. ** *p* < 0.05.

**Figure 14:**
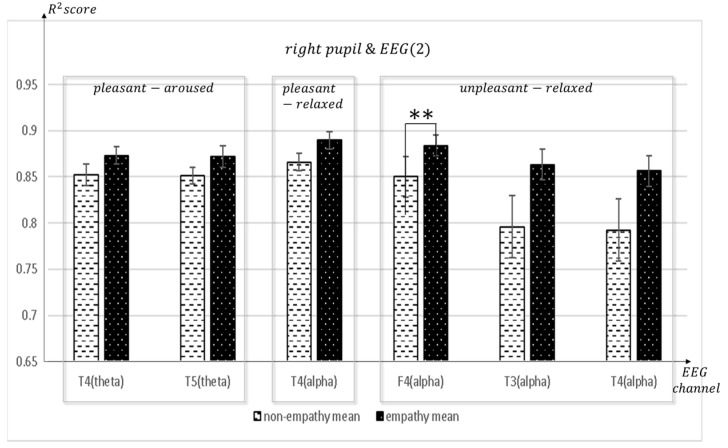
The mean value of the R^2^ score obtained by regression analysis of right pupil and EEG channels (T4, T5, F4, T3) in each frequency band when empathic states are compared with non-empathic states. ** *p* < 0.05.

**Figure 15:**
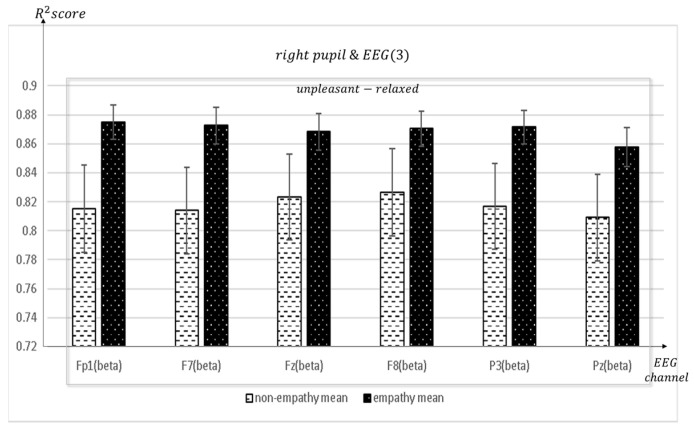
The mean value of the R^2^ score obtained by regression analysis of right pupil and EEG channels (Fp1, F7, Fz, F8, P3, Pz) in each frequency band when empathic states are compared with non-empathic states.

**Figure 16:**
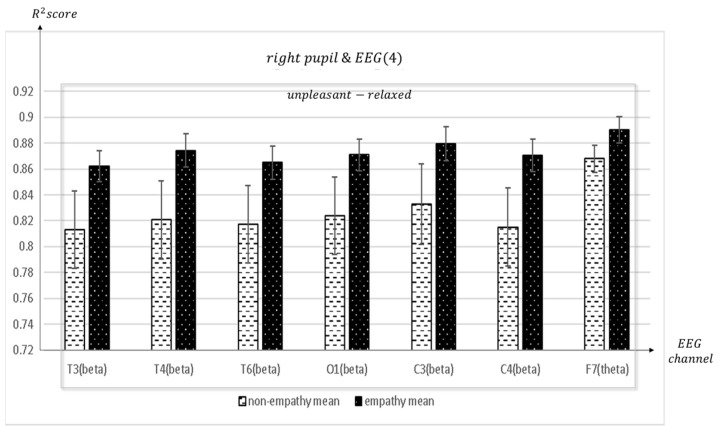
The mean value of the R^2^ score obtained by regression analysis of right pupil and EEG channels (T3, T4, T6, O1, C3, C4, F7) in each frequency band when empathic states are compared with non-empathic states.

**Figure 17:**
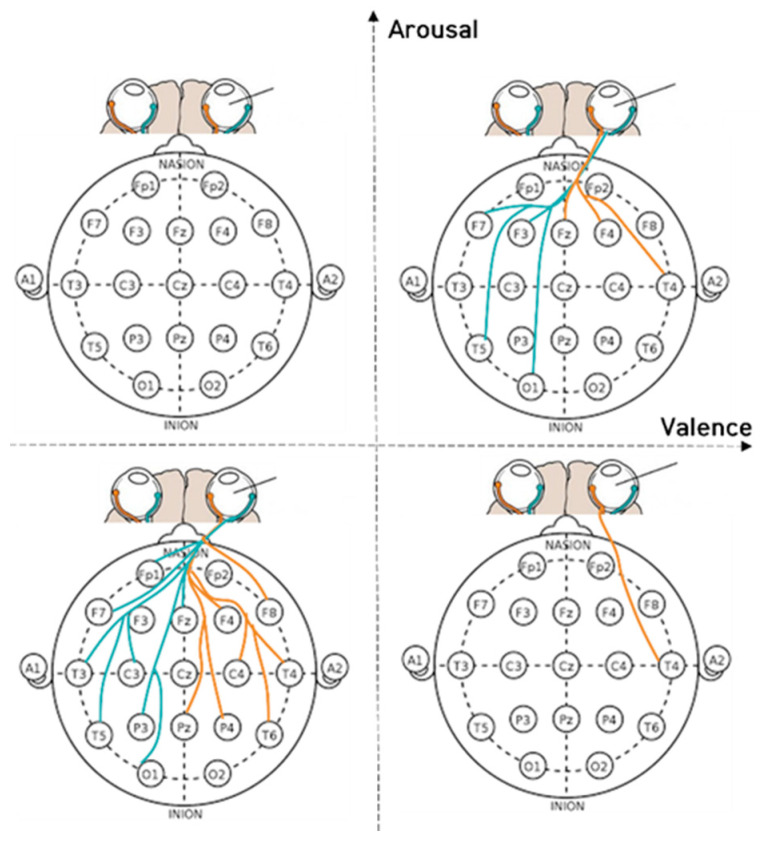
The right pupil and EEG channels in each frequency band in empathic states.

Figure 11, Figure 12, Figure 13, Figure 14, Figure 15, Figure 16 and Figure 17 shows that synchronization appears in the frontal lobe (Fp1 and left pupil: mean = 0.8773, std = 0.0123; F3 and left pupil: mean = 0.8864, std = 0.0105; Fz and left pupil: mean = 0.8819, std = 0.0101), parietal lobe (Pz and left pupil: mean = 0.8767, std = 0.0113), temporal lobe (T5 and left pupil: mean = 0.8814, std = 0.0086), and the left pupil of the eye when empathizing with pleasant-aroused content. However, synchronization with the frontal lobes (F3 and right pupil: mean = 0.8917, std = 0.0098; F4 and right pupil: mean = 0.8837, std = 0.0128; Fz and right pupil: mean = 0.8912, std = 0.0112; F7 and right pupil: mean = 0.877, std = 0.0103;), temporal lobe (T4 and right pupil: mean = 0.8733, std = 0.0097; T5 and right pupil: mean = 0.8719, std = 0.0116), occipital lobe (O1 and right pupil: mean = 0.8869, std = 0.0107), and the right pupil of the eye. When empathizing with the pleasant-relaxed content, synchronization appeared in the temporal lobe T4 (mean = 0.8895, std = 0.0093) and the right pupil of the eye.

When empathizing for unpleasant-relaxed, content appears in synchronization with many channels in the frontal F4 and right pupil: mean = 0.884, std = 0.011; Fp1 and right pupil: mean = 0.8751, std = 0.0118; F7 and right pupil: mean = 0.8727, std = 0.0127; Fz and right pupil: mean = 0.8684, std = 0.0126; F8 and right pupil: mean = 0.8704, std = 0.0122), parietal (P3 and right pupil: mean = 0.8715, std = 0.0115; Pz and right pupil: mean = 0.8577, std = 0.0134), temporal (T3 and right pupil: mean = 0.8633, std = 0.0168; T4 and right pupil: mean = 0.8564, std = 0.0165; T6 and right pupil: mean = 0.8649, std = 0.0128), occipital (O1 and right pupil: mean = 0.8709, std = 0.0123), and center (C3 and right pupil: mean = 0.8795, std = 0.0131) (C4 and right pupil: mean = 0.8705, std = 0.0125) and right pupils of the eye.

The synchronization between the EEG channel and the right pupil response is more than the left pupil response. Pupil changes are controlled by the central nervous system (oculomotor nerve) and autonomic nervous system (iris muscle). In synchronization between EEG and eye pupil change, viewers empathized with pleasant-aroused and unpleasant-relaxed videos, in which both cognitive and emotional empathy appeared.

### 3.3. Result of the Two-Dimension Emotional Model

**Figure 18:**
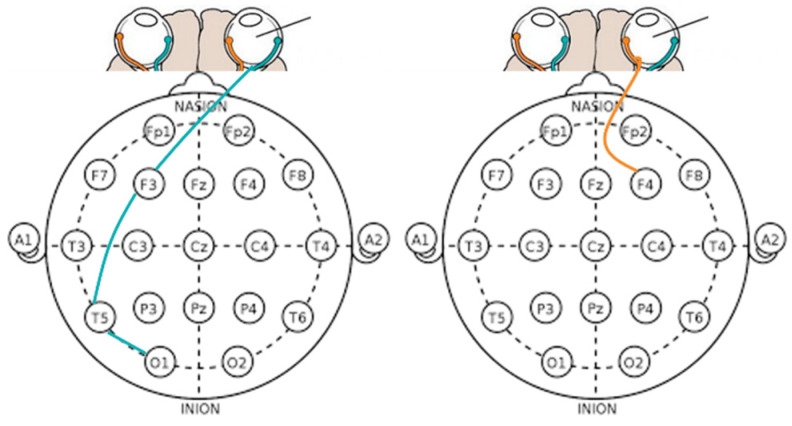
Pupil features (left pupil and right pupil) in eye movements and brain activity synchronization represent brainwave co-movements in a 2-dimensional emotional model.

Figure 18 illustrates that during pleasant empathy, the temporal lobe (T5) exhibited greater synchronization than in unpleasant empathy. This is attributed to the synchronization of the right pupil with F3, T5, and O1 when pleasant empathy is elicited (i.e., F3, T5, and O1 are interconnected).

This indicates that after visual information is transmitted from the left brain’s occipital lobe to the temporal lobe and subsequently to the frontal lobe for processing, the response interacts with the right pupil, culminating in the completion of visual processing cognition and the reaction associated with the pleasant empathy state.

Consequently, viewers perceive and pay more attention to pleasant content compared to unpleasant content. The pleasant condition video features company employees celebrating together, which encourages participants to engage in cognitive processing. In contrast, the unpleasant condition video portrays a mother’s grief upon learning of her daughter’s death, evoking a more emotional empathy response.

## 4. Conclusions

Empathy evocation leads to the activation of frontal and temporal lobes. The right cerebral hemisphere, which is associated with emotions involving social relationships, is where this response takes place. Specifically, the connectivity between the frontal and temporal lobes correlates with emotions triggered by social factors [25]. In our study, we selected 47 participants who viewed 8 emotionally charged videos. We monitored the participants’ brainwaves and eye movement data, and conducted subjective evaluations after each video session. The findings revealed the following: (1) Subjects were more likely to experience empathy while watching pleasant-aroused and negative-relaxed videos. (2) Brainwave and eye movement biometrics in the empathic state indicated that eye movement features such as saccades and fixations occurred synchronously with specific channels of the prefrontal and temporal lobes. (3) In the eigenvalues of brainwaves and pupil changes, the right pupil and select channels of the prefrontal, parietal, and temporal lobes exhibited synchrony during empathy. These results suggest that eye movements may serve as significant indicators of cognitive empathy towards videos, while the pupil might reflect the combined influence of emotional and cognitive empathy on video perception.

Previous research has found that connectivity between the right temporal and right occipital lobes is strongly associated with verbal memory, arousal, and the recollection of negative information. The corresponding socio-emotional responses exhibit both relational and positive–negative reactions (information-sharing for negative affective sharing and information-sharing for positive emotion). Connectivity between the left frontal and right parietal lobes, as well as between the left frontal and right parietal lobes, is demonstrated as connection responses based on the subject’s visual information. The related socio-emotional response is linked to emotion sharing and positive emotion sharing. These findings align with the outcomes of the current study.

Previous EEG studies exploring the neural correlates of empathy for emotions (pleasant-aroused, unpleasant-relaxed) have provided evidence of a shared neural circuit’s activation, stimulating synchronization between the viewer’s eye movement and the video’s physical attributes (target) [26,27]. This has been interpreted as an automatic and bottom-up processing component of empathy. In comparison, Zhang et al. investigated the relationship between brainwave connectivity and eye movement features within a 2-dimensional emotional model and discovered that the prefrontal, parietal, and temporal lobes are associated with cognitive activities [18], consistent with our findings. Future research on empathic videos may solely rely on eye movements and pupil data to determine the brain’s level of empathy.

Our study has some evident limitations. Firstly, we did not control and analyze the statistics of eye movements across different conditions, which could introduce confounding variables. Secondly, we cannot exclude the possibility that the observed effects may have been influenced by arousal modulation caused by the stimuli. Future research should properly control arousal to prevent this variable from causing confounding issues.

We would like to provide a comment regarding the EEG equipment utilized in this research. To accurately identify the location where bioelectrical signals are generated, it is necessary to use high-density recording electrodes. Therefore, we intentionally opted for 18 channels and a high sampling frequency of 500 Hz. Although there are simpler EEG devices available, we caution against using them for scientific research due to their limited spatial resolution (such as the MUSE with only four electrodes) and channel capacity (such as the Neurosky with a single channel). While the EMOTIV has 14 channels, its signal quality is average.

## Figures and Tables

**Table 1:** Questionnaire of empathy, valence, and arousal.

NO.	Questionnaire	Factor
1	I felt pleasant as opposed to unpleasant	Valence
2	I felt aroused as opposed to relaxed	Arousal
3	I understood the characters’ needs	Cognitive empathy
4	I understood how the characters were feeling	
5	I understood the situation of the video	
6	I understood the motives behind the characters’ behavior	
7	I felt as if the events in the video were happening to me	Affective empathy
8	I felt as if I was in the middle of the situation	
9	I felt as if I was one of the characters	

**Table 2:** Results of regression prediction. The number indicates the training iterations.

	Number	0	1	2	3	4
Model						
Bayesian Ridge		−4.60010	−3.71430	−5.99860	−2.44218	−14.07775
Linear Regression		−4.24814	−3.74845	−6.25194	−2.44820	−14.00138
Elastic Net		−6.66114	−3.64038	−2.25533	−3.63111	−15.92946
SVR		−9.87530	−4.16286	−3.07216	−0.89799	−16.68947
GBR		−3.59616	−8.14732	−3.177	−4.73490	−15.46454

**Table 3:** The model of evaluation indicators.

	Indicator	EV	MAE	MSE	R^2^-Score
Model					
Bayesian Ridge		1.48 × 10^−1^	0.02106	0.00065	0.14844
Linear Regression		1.49 × 10^−1^	0.02105	0.00065	0.14866
Elastic Net		0.00	0.02321	0.00076	0
SVR		−2.22 × 10^−16^	0.02456	0.00079	−0.04318
GBR		8.26 × 10^−1^	0.00894	0.00013	0.82640

## Data Availability

Not applicable.
